# Sjögren-Larsson syndrome in two brothers: a case report

**DOI:** 10.4076/1757-1626-2-8434

**Published:** 2009-09-09

**Authors:** Farid Rezaei Moghaddam, Farid Safar, Mahsa Asheghan, Zahra Reza Soltani, Fatemeh Dehghani Zade

**Affiliations:** 1 Department of Physical Medicine and Rehabilitation, Army University of Medical Sciences, 501 HospitalEtemadzadeh Street, Western Fatemi Street, TehranIran; 2 Department of Dermatology, Tehran University of Medical Sciences, Imam Khomeini HospitalGharib street, TehranIran; 3 Department of Physical Medicine and Rehabilitation, University of Welfare and RehabilitationTehranIran; 4 Department of Physical Medicine and Rehabilitation, Semnan University of Medical SciencesTehranIran

## Abstract

Sjögren-Larsson syndrome is a rare autosomal recessive disorder that was originally recognized in the coexistence of congenital ichthyosis, spastic diplegia or quadriplegia and mental retardation. We recently saw two cases with characteristic features of this rare syndrome. Two brothers aged 21 and 25 years presented with triad of congenital ichthyosis, mental retardation and spastic diplegia. Magnetic resonance imaging showed demyelinating disease in one of these cases. Electrodiagnostic studies were normal in all cases.

## Introduction

Sjögren-Larsson syndrome (SLS) is a rare neurocutaneous disorder. Additional clinical findings have been reported include characteristic retinopathy, pruritus, preterm birth and skeletal abnormality [1]. SLS is due to a genetic block in the oxidation of fatty alcohol to fatty acid because of deficient activity of fatty aldehyde dehydrogenase (FALDH). This enzyme catalyzes the oxidation of long chain fatty aldehydes to fatty acids. Accumulation of long-chain fatty alcohols and modification of macromolecules by an excess of fatty aldehydes are thought to be the pathophysiologic mechanisms causing the manifestations of SLS [2]. It occurs in all races and its prevalence has been estimated as 0.4 per 100,000 or lower. Over 200 cases worldwide have been reported [3]. Here we describe two cases in an Iranian family.

## Case presentation

**Figure 1.**
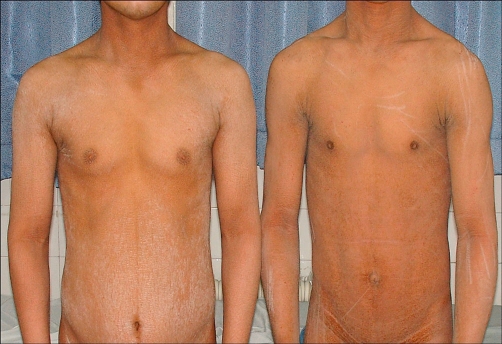
Photograph of the highly characteristic abnormalities of the skin.

Two brothers aged 21 and 25 years from Iranian family, presented with generalized dryness of skin since birth and difficulty in walking since the age of 4-5 years. There was a history of consanguinity in parents. The parents and siblings were healthy, but their cousin was affected by self-report. The younger brother was delivered at full term by normal vaginal delivery and the elder brother was born pre-term. Skeletal abnormalities (e.g. short stature, kyphoscoliosis) were not observed. They were not performing well at school. Cutaneous examination revealed generalized dryness of skin with fine scales most predominant around the umbilicus and in the flexural folds (Figure 1). Neurological examination revealed moderate mental retardation in both of them. Spasticity was found in two brothers, it involved the legs and presented since the age of 5-6 years. Their gait was spastic with brisk deep tendon reflexes in the lower limbs. The patients had grade 4/5 power in both lower limbs. These features were more marked in the younger brother, as were the cutaneous findings. Upper limbs had normal power, tone and deep tendon reflexes. There were no sensory, extrapyramidal or cerebellar signs in our cases. Sensory exam included test for light touch, superficial pain, temperature, position sense and vibration. Evaluation of cerebellar function (finger-nose, heel-shin, Romberg test, dysdiadochokinesia) and extrapyramidal exam (smoothness of motor function) was normal.

**Figure 2.**
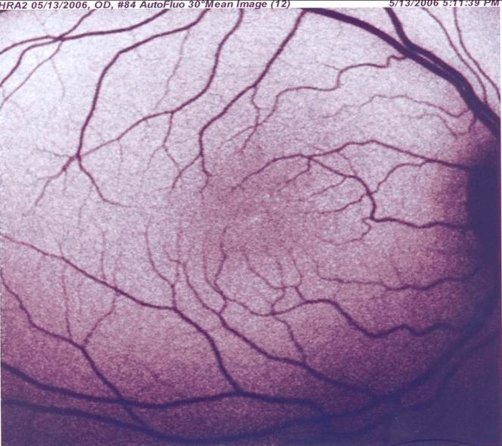
Fluorescein angiography at the left eye of younger brother, including macular Glistening dot.

**Figure 3.**
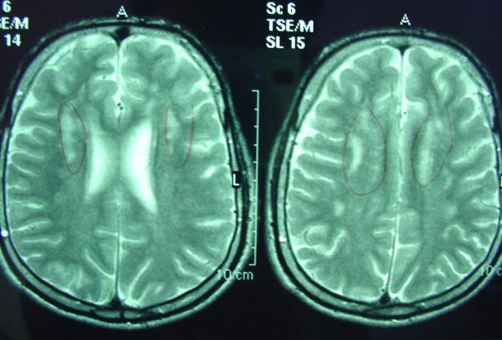
T2-weighted MR images. Signal-intensity changes of the periventricular white matter with involvement of the frontal trigones.

Pruritus was an accompanying disabling feature in patients. Skin biopsy showed orthohyperkeratosis, acanthosis and papillomatosis. An ocular examination revealed bilateral Glistening dot on the macular region of the retina in both of them (Figure 2). In elder brother, brain MRI revealed dysmyelination in the deep periventricular white matter and reduced brain volume in frontal lobe (Figure 3). The younger brother had normal brain MRI. Nerve conduction study and electromyography were normal in cases. All EEG recordings showed symmetrical slow background activity without other abnormalities. The histochemical analysis of hexonal dehydrogenase in skin and biochemical assay of leukocytes or fibroblasts for FALDH were not available. The lipid profile was normal.

## Discussion

SLS is an inborn error of fatty alcohol oxidation. Very high prevalence of SLS has been observed in north east of Sweden where an incident of 8.3:100,000 births has been reported [4]. However, there are no epidemiological reports on its frequency in the Iranian population, and as far as we know, these are the first cases of SLS with pathognomonic findings in Iran. In our patients, the signs and symptoms were nonspecific and physicians did not consider SLS as a diagnosis. The diagnosis of SLS is almost always delayed because usually only cutaneous symptoms are present at birth. Newborns usually manifest symptoms and signs of the disease (first ichthyosis, subsequently neurologic symptoms). Since the skin disorder is the most prominent sign in infancy, patients were first seen by dermatologist and minor neurologic signs were missed. The diagnosis was made later, when neurologic signs appeared. Spasticity may be apparent before age 3 years and is more severe in the lower limbs than in other parts of the body [5]. After neurologic symptoms appear, development is progressively delayed. No progression of the neurologic findings or mental retardation occurs after puberty. The diagnosis should be considered in all patients with generalized hyperkeratosis and central nervous system dysfunction. The presence of glistening dots in the macular region is considered pathognomonic, although it is not constant. The nature of the crystalline deposits in the retina is unclear. It is speculated that they might represent accumulations of long-chain fatty alcohols or fatty aldehydes [6]. This finding was present in our cases. About 30-60% of patients have convulsion during infancy. This was not the case in our patients. MR imaging shows retardation of myelination and dysmyelination.

A clear relationship between the degree of the MRI abnormality and the neurological features or the age of patients could not be demonstrated. In SLS, MR imaging abnormalities of the brain are confined to the cerebral white matter and the corticospinal tracts. They consist of the accumulation of lipid substrates, delayed myelination, periventricular gliosis, and a permanent myelin deficit. The patterns are strikingly similar among patients with SLS, but the severity varies [7,9]. The differential diagnosis of SLS is other neuroichthyotic disorders and confirmed by the very low level of FALDH activity. However, this test is not available in our country. The clinical triad of syndrome eliminates the possibilities of other ichthyosiform erythrodermas with neurologic signs. In atypical cases, the differential diagnosis might encompass peroxisomal disorders (e.g. Refsum disease), and many other rare syndromes which involve multiple organs [10]. It is useful to evoke the diagnosis when spastic paraparesis is associated with unusual cutaneous signs.

Management in SLS is supportive [11]. Topical moisturizing lotion, keratolytic agents and oral retinoids was administered as outpatient medications. The daily management of the ichthyosis was taught. Training to provide home-based physical therapy is useful to prevent contractures. Stretching of muscles in lower limbs and aquatic therapy was administered for patients. Presently, our patients have no progression of symptoms. Spasticity in lower limbs has decreased and they have remained ambulatory without assistance. The elder brother has married and has a healthy daughter. Also, cutaneous symptoms were improved after treatment.

## Abbreviations

EEG: electroencephalogram
FALDH: fatty aldehyde dehydrogenase
MRI: magnetic resonance imaging
SLS: Sjögren-Larsson syndrome
